# Dicer1 Ablation in the Mouse Epididymis Causes Dedifferentiation of the Epithelium and Imbalance in Sex Steroid Signaling

**DOI:** 10.1371/journal.pone.0038457

**Published:** 2012-06-06

**Authors:** Ida Björkgren, Lauri Saastamoinen, Anton Krutskikh, Ilpo Huhtaniemi, Matti Poutanen, Petra Sipilä

**Affiliations:** 1 Department of Physiology, Institute of Biomedicine, University of Turku, Turku, Finland; 2 Turku Graduate School of Biomedical Sciences, Turku, Finland; 3 Institute of Reproductive and Developmental Biology, Imperial College London, Hammersmith Campus, London, United Kingdom; 4 Turku Center for Disease Modeling, (TCDM), University of Turku, Turku, Finland; Clermont-Ferrand Univ., France

## Abstract

**Background:**

The postnatal development of the epididymis is a complex process that results in a highly differentiated epithelium, divided into several segments. Recent studies indicate a role for RNA interference (RNAi) in the development of the epididymis, however, the actual requirement for RNAi has remained elusive. Here, we present the first evidence of a direct need for RNAi in the differentiation of the epididymal epithelium.

**Methodology/Principal Findings:**

By utilizing the Cre-LoxP system we have generated a conditional knock-out of Dicer1 in the two most proximal segments of the mouse epididymis. Recombination of *Dicer1*, catalyzed by *Defb41^iCre/wt^*, took place before puberty, starting from 12 days postpartum. Shortly thereafter, downregulation of the expression of two genes specific for the most proximal epididymis (lipocalin 8 and cystatin 8) was observed. Following this, segment development continued until week 5 at which age the epithelium started to regress back to an undifferentiated state. The dedifferentiated epithelium also showed an increase in estrogen receptor 1 expression while the expression of androgen receptor and its target genes; glutathione peroxidase 5, lipocalin 5 and cysteine-rich secretory protein 1 was downregulated, indicating imbalanced sex steroid signaling.

**Conclusions/Significance:**

At the time of the final epididymal development, Dicer1 acts as a regulator of signaling pathways essential for maintaining epithelial cell differentiation.

## Introduction

After development in the testis, the spermatozoa travel through the epididymis where they mature, gaining motility and the ability to fertilize the oocyte. Despite consisting of a single long duct, the epididymis is a complex organ, highly convoluted and divided into several anatomical and functional segments, i.e. initial segment (IS), caput (CAP), corpus (COR) and cauda (CAU). Each segment synthesizes and secretes a specific set of proteins, thus creating the unique luminal environment needed for the sperm maturation process [1]–[3]. The most proximal segments (IS and CAP) have been proven essential for sperm maturation as the disruption of their development or function often leads to male infertility [4]–[7]. The epididymis develops from the mesonephric tubules and the proximal Wolffian duct (WD) [8]. During the embryonic stage, and before epithelial differentiation, mesenchymal androgen receptor (AR), along with inhibin beta A (Inhba), facilitates the elongation and convolution/coiling of the tubule [9]–[11]. Development of the epididymis continues after birth with differentiation of the epithelial cells into principal, basal and narrow/clear cells [2], [2], [12,12,13]. At the onset of spermatogenesis, the epididymal epithelium develops segment-specific gene expression [14]. From studies with genetically modified mice, it has become evident that leucine-rich repeat-containing G protein-coupled receptor 4 (LGR4, also known as GPR48) is needed for the postnatal epididymal coiling and the differentiation of IS [15], [16]. In addition, the proto-oncogene Ros1 (ROS1, also known as c-ros) is necessary for the formation of IS [5]. Recent studies with epididymal AR knock-out mice have revealed that androgen signaling is required for the formation of IS and differentiation of principal and basal cells [6], [7], [10]. However, there are still many unresolved issues regarding the regulatory pathways responsible for differentiation of the epididymal segments and their specific gene expression.

Many aspects of development are regulated by RNA interference (RNAi). Small non-coding RNAs bind to complimentary mRNA sequences and cause translational silencing and mRNA cleavage [17]. One class of small non-coding RNAs is the microRNAs (miRNAs) which are initially produced as longer precursors that need to be processed by the RNaseIII enzyme Dicer1 to become fully functional [18]. The ∼22 nt-long mature miRNAs then control protein expression and modulate diverse cellular events such as differentiation, proliferation, apoptosis and cell metabolism [19], [20]. Consequently, Dicer1 deficient mice die already on embryonic day 7.5 owing to complete loss of pluripotent stem cells [21]. To study the effect of RNAi in the development and function of specific organs, *Dicer1* conditional knock-out (*Dicer1* cKO) mice can be used. The need for Dicer1 in the differentiation of several cell types, including sensory epithelial cells, T cells and pancreatic β-cells, has been shown in *Dicer1* cKO mice by crossing *Dicer1^fl/fl^* mice with those expressing *Cre* in the cells of interest [12], [22]–[24]. Furthermore, a requirement for Dicer1 in the patterning of the liver and colon was also confirmed with *Dicer1* cKO mice. Hepatocyte specific Dicer1 ablation compromises region-specific protein expression while Dicer1 depletion in the developing colon leads to a disorganized epithelium [25], [26].

Previous studies on human and rat epididymides have shown that miRNAs are differentially expressed at juvenile and adult stages [27], [28], indicating a role for miRNAs in the postnatal development of the epididymis. To study the function of RNAi in the developing epididymis we generated epididymis-specific *Dicer1* cKO mice by crossing *Dicer1^fl/fl^* mice with *Defb41^iCre^* mice. This resulted in the elimination of *Dicer1* expression from the pre-pubertal epididymis. Our results demonstrate the importance of Dicer1 in sex steroid signaling and in maintenance of the differentiated state of the epididymal epithelium.

## Results

### Generation of *Dicer1^fl/fl^; Defb41^iCre/wt^* Mice

**Figure 1:**
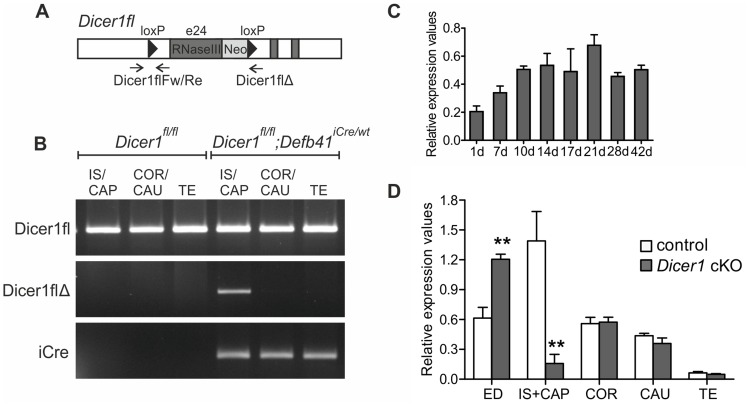
The epididymis specific *Dicer1* knock-out. (A) Schematic diagram of the *Dicer1* locus with loxP sites flanking exon 24 (e24). Arrows indicate the location of genotyping primers used for analyzing the deletion of e24. (B) Genomic PCR of 12 day-old mice showing the intact locus (Dicer1fl) and the recombinant locus with e24 deleted (Dicer1flΔ). Exon 24 is only deleted in the *Dicer1^fl/fl^; Defb41^iCre/wt^* mouse initial segment (IS) and caput (CAP) while the iCre locus is detected in all segments of the epididymis. (C) Expression of *Dicer1* mRNA in the whole epididymis of 1–42 day-old wild-type mice. (D) *Dicer1* mRNA expression levels in the efferent ducts (ED), the different segments of the epididymis and testis (TE) of 2 month-old control and *Dicer1* conditional knock-out (cKO) mice. Expression levels are presented relative to *Ppia* (testis) and *L19* (epididymis) expression. COR, corpus; CAU, cauda. Statistical significance was calculated from the expression levels of 3 control and 4 *Dicer1^fl/fl^; Defb41^iCre/wt^* mouse samples using the unpaired t-test. Statistical significance of changes is indicated as follows: **, *P≤0.01*. Schematic picture modified from Harfe et al., 2005 [60].

Quantitative RT-PCR of the mouse epididymis showed a continuous expression of *Dicer1* from birth into adulthood (Figure 1C). As the full knock-out of *Dicer1* is embryonically lethal [21], we generated a *Dicer1* cKO mouse line by crossing *Dicer1^fl/fl^* mice [29] with a mouse line expressing *iCre* under the Defensin beta 41 (*Defb41*) promoter. The *Dicer1^fl^* allele consists of two loxP sites flanking exon 24, which contains a major part of the second RNaseIII domain (Figure 1A). The heterozygous *Defb41^iCre^* mouse line did not show any phenotypic defects or fertility problems and expressed *iCre* in the epithelium of the most proximal part of the epididymis, IS and CAP. Recombination of *Dicer1* was observed in 12 day-old *Dicer1^fl/fl^; Defb41^iCre/wt^* mouse IS and CAP by genomic PCR (Figure 1B) and qRT-PCR studies revealed a significant reduction in *Dicer1* expression levels at the age of 2 months (Figure 1D).

### Morphology of the Epididymis and Fertility of *Dicer1^fl/fl^; Defb41^iCre/wt^* Males

**Figure 2:**
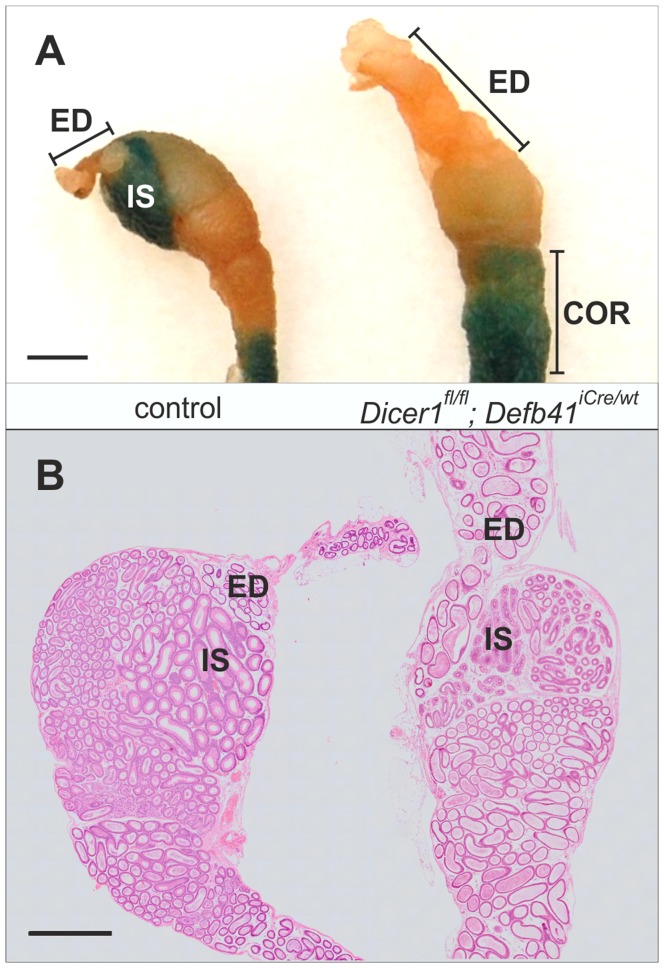
Morphology of 2 month-old mice proximal epididymides. (A) X-gal staining detecting endogenous β-galactosidase activity in a control mouse initial segment (IS) and proximal corpus (COR). *Dicer1^fl/fl^; Defb41^iCre/wt^* mice display no staining of initial segment and have enlarged efferent ducts (ED). (B) HE staining of the efferent ducts and the proximal epididymis of control and *Dicer1^fl/fl^; Defb41^iCre/wt^* mice. Scale bars 1.5 mm.

**Figure 3:**
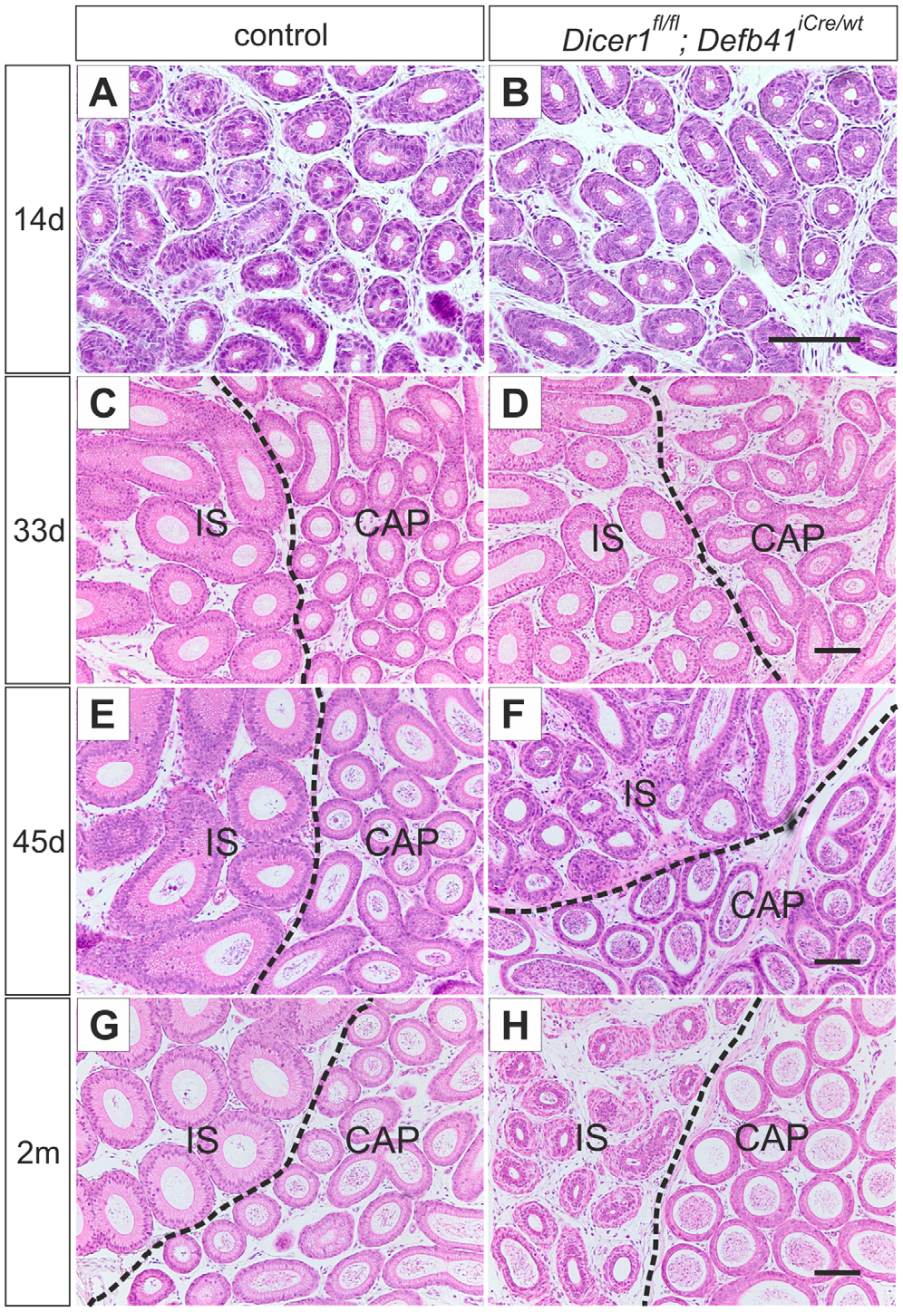
Differentiation of the epididymal epithelium. Hematoxylin and eosin staining of control and *Dicer1^fl/fl^; Defb41^iCre/wt^* mouse epididymides (A, B) The undifferentiated epithelium of the proximal epididymis of a 14 day-old control and a *Dicer1^fl/fl^; Defb41^iCre/wt^* mouse. (C, D) 33 day-old control and *Dicer1^fl/fl^; Defb41^iCre/wt^* mouse showing initiated differentiation of the initial segment (IS). (E) The fully developed IS of a 45 day-old control mouse. (F) The epithelium of a *Dicer1^fl/fl^; Defb41^iCre/wt^* mouse IS resembling that of the 14 day-old mouse. (G, H) The epididymis of an adult, 2 month-old, control and *Dicer1^fl/fl^; Defb41^iCre/wt^* mouse. CAP, caput. Scale bars 100 µm.

Macroscopic evaluation of 2 month-old *Dicer1^fl/fl^; Defb41^iCre/wt^* mice epididymides revealed an underdeveloped IS and, in addition, the mice frequently presented with enlarged efferent ducts (Figure 2A, B). The IS of control mice can be clearly visualized owing to the endogenous β-galactosidase activity in the segment. The much smaller IS of *Dicer1^fl/fl^; Defb41^iCre/wt^* mice could not be distinguished from CAP with X-gal staining (Figure 2A). Furthermore, the intense vasculature typical of WT IS was missing from the *Dicer1* cKO IS. Histological evaluation showed a division of the epididymis into different segments (Figure 2B) but the epithelial cell layer of both IS and CAP was disorganized (Figure 3H). *Dicer1^fl/fl^* mice have a similar phenotype to WT mice epididymides and were used as controls throughout the study. *Dicer1* cKO epididymides were significantly smaller than those of control mice (30.4±1.5 mg, control: 35.4±0.7 mg, *P≤0.01*). No significant difference in the weight of 6 month-old *Dicer1* cKO and control mice epididymides was observed. However, the epithelial cell layer of the 6 month-old *Dicer1^fl/fl^; Defb41^iCre/wt^* mouse was further disturbed, with neoplastic changes in the efferent ducts causing their progressive obstruction (Figure S1). Even though sperm were detected in the CAU, 2- to 3-month-old *Dicer1^fl/fl^; Defb41^iCre/wt^* male mice failed to produce offspring when mated with WT females (Table 1). The number of sperm was reduced in *Dicer1^fl/fl^; Defb41^iCre/wt^* mouse epididymides as histological staining showed some tubular cross sections with no sperm. At 6 months of age the number of tubular cross sections without sperm was further increased due to the obstruction of the *Dicer1^fl/fl^; Defb41^iCre/wt^* mouse efferent ducts. The testis of the 6 month-old *Dicer1^fl/fl^; Defb41^iCre/wt^* mouse also displayed disruption of the seminiferous epithelium owing to fluid back-pressure (data not shown). Further morphological analyses revealed that the number of sperm with angulated tails was not significantly increased in 2 month-old *Dicer1^fl/fl^; Defb41^iCre/wt^* mouse CAU (22.9±3.4% of all sperm, control: 16.3±1.2%).

**Table 1:** Fertility of *Dicer1^fl/fl^; Defb41^iCre/wt^* male mice.

	No. of Males	Total No. of Copulatory Plugs	Total No. of Litters	Average No. of Pups per Litter
control	3	13	12	9.2
*Dicer1^fl/fl^; Defb41^iCre/wt^*	5	20	0	0

### Effects of *Dicer1* cKO on Epididymal Cell Differentiation

At the age of one month, the IS of both control and *Dicer1^fl/fl^; Defb41^iCre/wt^* mice was distinguishable from the CAP by its high, columnar-shaped epithelial cells (Figure 3C, D). However, the height of the epithelium of *Dicer1* cKO IS was reduced below that of control mouse IS. In the 45 day-old mouse, the epithelial cells of the *Dicer1* cKO IS had regressed to an undifferentiated state with a disorganized epithelial cell pattern (Figure 3E, F), resembling those of the epididymis of 14 day-old mice (Figure 3A, B). Furthermore, the height of the epithelial cells of IS was significantly reduced (average cell height in control IS: 45 µm, in *Dicer1* cKO IS: 28 µm, *P≤0.0001*) in 2 month-old *Dicer1^fl/fl^; Defb41^iCre/wt^* mice. However, the phenotype of the *Dicer1* cKO IS varied between individuals from a severely disorganized epithelial cell layer to a thin epithelial cell layer resembling that of COR (Figure S2) in some individuals.

**Figure 4:**
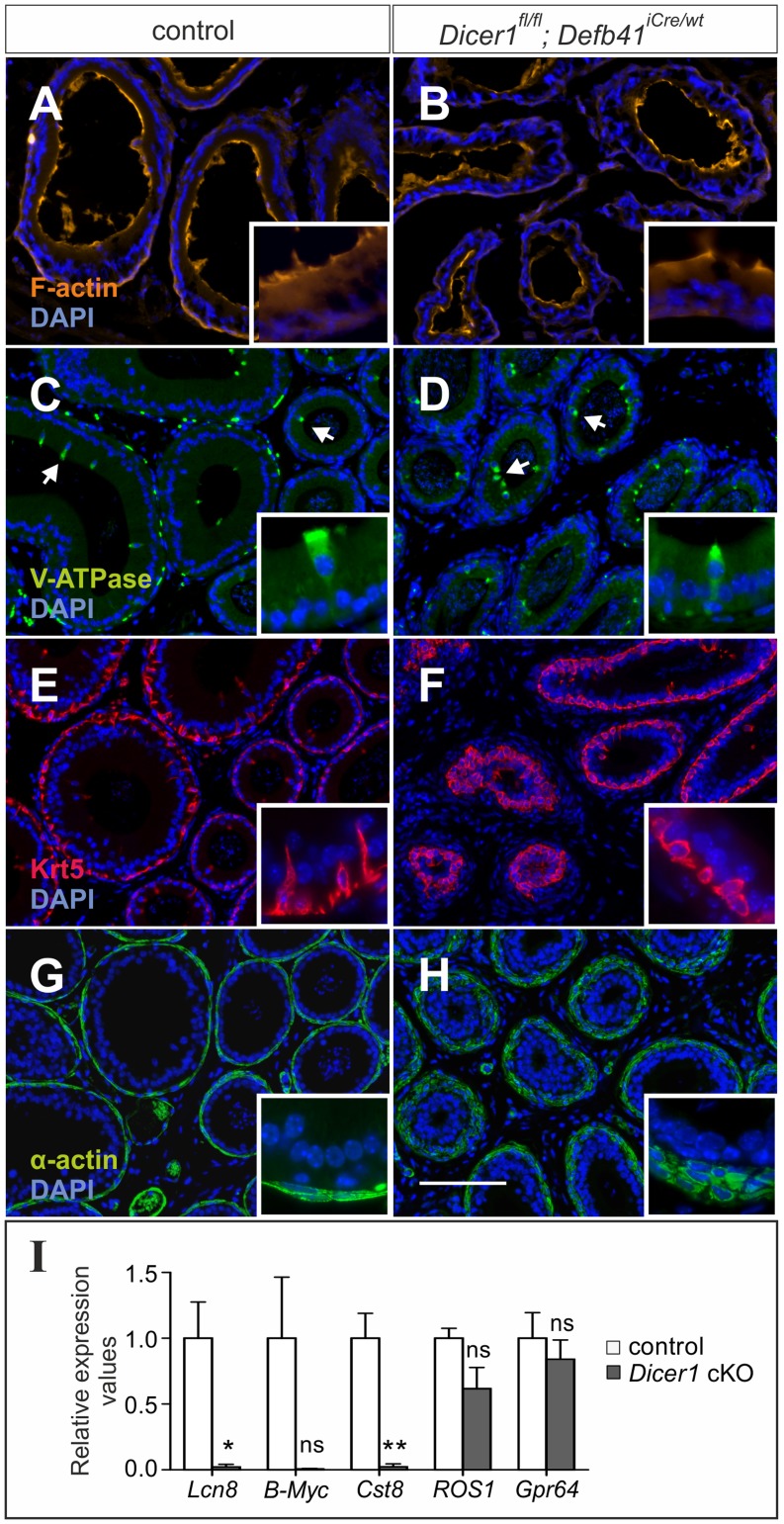
Immunohistochemical staining of the different epithelial cell types. Staining of initial segment (A, B) and initial segment and caput (C–H) of two-month-old control and Dicer1fl/fl; Defb41iCre/wt mouse epididymides. (A, B) Staining of principal cell F-actin by phalloidin-TRITC. (C, D) Expression of vacuolar H+-ATPase (V-ATPase) in narrow and clear cells (marked with arrows). (E, F) Expression of keratin 5 (Krt5) in basal cells. (G, H) Smooth muscle cells. Dicer1fl/fl; Defb41iCre/wt mice display a thicker smooth muscle cell layer than that of control mice. Inserts are 3× enlargements of the stained cells. (I) qRT-PCR for proximal epididymis-specific gene expression. Expression of lipocalin 8 (Lcn8), brain expressed myelocytomatosis oncogene (Bmyc), cystatin 8 (Cst8), Ros1 proto-oncogene (Ros1) and G protein-coupled receptor 64 (Gpr64) in the initial segment and caput of 2 month-old control and Dicer1 conditional knock-out (cKO) mouse epididymides. Values relative to L19 expression. Statistical significance was calculated from the expression levels of 3 control and 3 Dicer1fl/fl;Defb41iCre/wt mouse (Ros1∶8 control and 9 Dicer1fl/fl;Defb41iCre/wt mouse) samples using the unpaired t-test. Statistical significance of changes is indicated as follows: ns, not significant; *, P≤0.05; **, P≤0.01. Scale bars 100 µm.

To study further the effect of *Dicer1* ablation on epithelial cell differentiation, the presence of different epithelial cell types of the epididymal epithelium was analyzed by immunohistochemistry. Phalloidin conjugated to TRITC was used to stain the F-actin of principal cells, and antibodies against vacuolar H^+^-ATPase (V-ATPase) and Keratin 5 (Krt5) were used to stain clear/narrow cells and basal cells, respectively. The results indicated that all cell types were present in the *Dicer1* cKO epididymis (Figure 4). The epithelial cell types were found in similar numbers and locations in the *Dicer1* cKO epithelium as in control animals. However, α-actin staining revealed an increase in muscle cell layer thickness (Figure 4G, H). The *Dicer1^fl/fl^; Defb41^iCre/wt^* mice displayed three layers of muscle cells surrounding the epididymal duct, whereas control mice typically had one.

Genes specifically expressed in the mature proximal epididymis, IS and proximal CAP; lipocalin 8 (*Lcn8*), brain expressed myelocytomatosis oncogene (*Bmyc*) and cystatin 8 (*Cst8*), showed a marked downregulation in their expression in IS and CAP of 2 month-old *Dicer1^fl/fl^; Defb41^iCre/wt^* mice. The only exceptions were *Ros1* and G protein-coupled receptor 64 (*Gpr64*, also known as *HE6*) that showed no significant difference in expression levels compared with the control (Figure 4I). The expression of *Cst8*, *Lcn8* and *Ros1* is initiated at postnatal days 20, 21 and 16, respectively [5], [30], [31]. The qRT-PCR results from different time points during epididymal development showed significantly reduced expression of both *Cst8* and *Lcn8* in *Dicer1^fl/fl^; Defb41^iCre/wt^* mice, from 21 days postpartum onward (Figure S3A, B). However, the expression levels of *Ros1* did not differ significantly from that of control mice (Figure S3C).

### Cell Proliferation and Apoptosis

**Figure 5:**
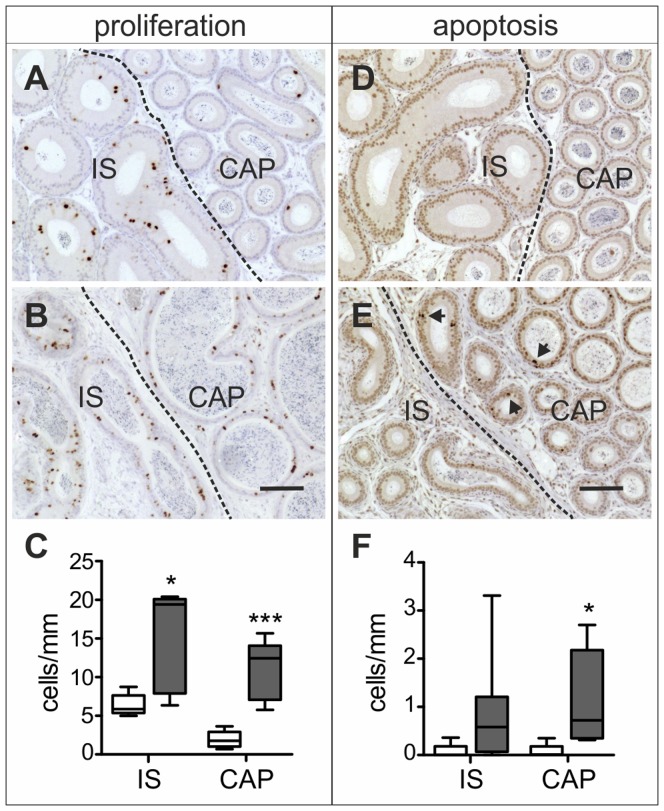
Cell proliferation and apoptosis. Ki67 immunostaining; (A) Control; (B) *Dicer1^fl/fl^; Defb41^iCre/wt^* mouse. TUNEL labeling; (D) Control; (E) *Dicer1^fl/fl^; Defb41^iCre/wt^* mouse. Arrows mark apoptotic cells. (C, F) Comparison of number of proliferating cells and apoptotic cells in the initial segment (IS) and caput (CAP) of control (white bars) and *Dicer1^fl/fl^; Defb41^iCre/wt^* mice (grey bars). The number of proliferating and apoptotic cells were calculated from ten tubular cross sections of 5 control and 5 (Ki67 stained) and 8 (TUNEL labeled) *Dicer1^fl/fl^;* *Defb41^iCre/wt^* mice epididymides and the total cell number was divided by the circumference of the tubular cross sections (mm). Statistical significance of changes, calculated using the unpaired t-test, is indicated as follows: ns, not significant; *, *P≤0.05*; ***, *P≤0.001*. Scale bars 100 µm.

Immunohistochemical studies showed a marked increase in cell proliferation throughout the proximal epididymis of 2 month-old *Dicer1^fl/fl^; Defb41^iCre/wt^* mice (Figure 5B). The number of Ki-67 positive cells was on average 2 times higher in *Dicer1* cKO IS (15.2±3.0 cells/mm, control: 6.4±0.6 cells/mm, *P≤0.05*) and almost 6 times higher in *Dicer1* cKO CAP (11.1±1.7 cells/mm, control: 1.9±0.5 cells/mm, *P≤0.001*) compared with those of control mice epididymides (Figure 5C). Although the epithelium was highly proliferative, it did not lead to a marked increase in the size of the *Dicer1^fl/fl^; Defb41^iCre/wt^* mouse epididymides. On the contrary, at the age of 2 months the *Dicer1* cKO epididymis was significantly smaller than that of the control mouse. Furthermore, the number of apoptotic cells was increased in *Dicer1* cKO IS and CAP (*Dicer1* cKO IS: 0.89±0.39 cells/mm, control IS: 0.07±0.07 cells/mm; *Dicer1* cKO CAP: 1.12±0.34 cells/mm, control CAP: 0.07±0.07 cells/mm, *P≤0.05*) (Figure 5D–F). The difference seen in IS was not statistically significant owing to high variation between the individual mice.

### Expression of Sex Steroid and Fibroblast Growth Factor Receptors in the *Dicer1* cKO IS and CAP

To study whether the observed epididymal phenotype is caused by changes in sex steroid signaling, the expression of *Ar,* estrogen receptor 1 (*Esr1*, also known as *ERα*) and estrogen receptor 2 (*Esr2*, also known as *ERβ*) was analysed by qRT-PCR and immunohistochemistry in 2 month-old *Dicer1* cKO and control epididymides. The qRT-PCR results showed a significantly weaker expression of *Ar* in *Dicer1* cKO IS and CAP (Figure 6E), and downregulation of AR target gene expression, cysteine-rich secretory protein 1 (*Crisp1*), glutathione peroxidase 5 (*Gpx5*) and lipocalin 5 (*Lcn5*), was also observed (Figure 6F). *Esr2* showed a similar reduction in mRNA expression levels as *Ar*, while the expression of *Esr1* did not significantly differ between *Dicer1* cKO and control IS and CAP (Figure 6E). However, immunohistological staining displayed an altered ESR1 expression pattern in *Dicer1* cKO IS. ESR1 was foremost expressed in the narrow cells of control mouse IS, whereas the *Dicer1* cKO IS showed equal expression of the receptor in almost all epithelial cells (Figure 6A, B). Furthermore, immunohistochemical analyses clearly showed weaker AR staining in a number of the *Dicer1* cKO epididymides analysed (Figure 6D). Nevertheless, the AR findings were variable and some *Dicer1* cKO epididymides had an AR staining equal to that of the controls. Furthermore, the relative expression values of fibroblast growth factor receptors 1–4 (*Fgfr1*–*4)* were not statistically different when comparing qRT-PCR results from *Dicer1^fl/fl^; Defb41^iCre/wt^* mice with control mouse IS and CAP (data not shown).

**Figure:**
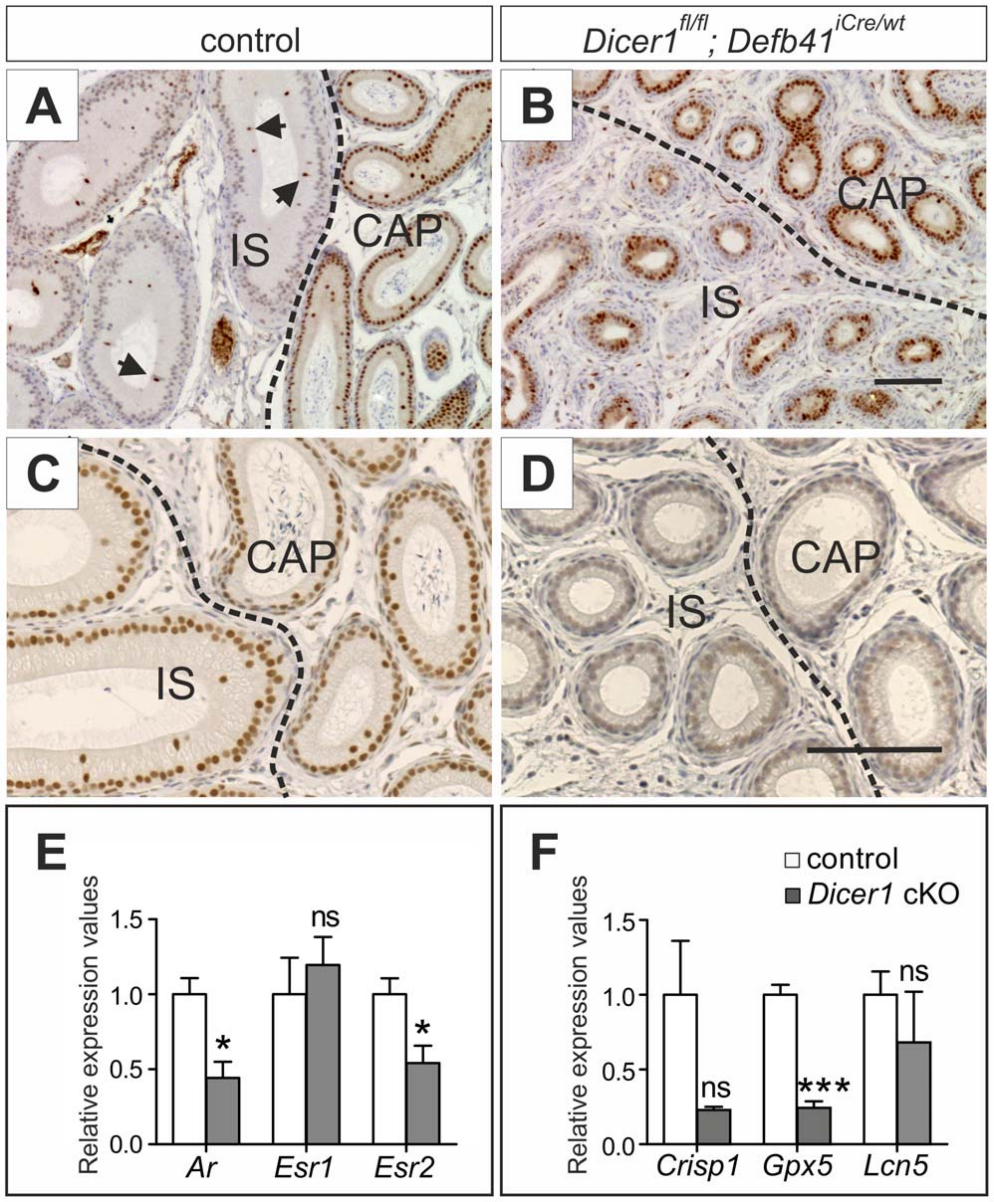
Expression of Sex steroid receptors. (A) Immunostaining of two-month-old control mouse initial segment (IS) shows expression of Estrogen receptor 1 (ESR1) only in the narrow cells (indicated by arrows) while (B) the *Dicer1^fl/fl^; Defb41^iCre/wt^* mouse has ESR1 expression in most cells of the epithelium. (C, D) Staining for Androgen receptor. (D) Androgen receptor expression varied between *Dicer1^fl/fl^; Defb41^iCre/wt^* mice epididymides. Some samples had similar expression levels as the control while others showed areas of more weakly stained tissue. CAP, caput. Scale bars 100 µm. (E) Expression of androgen receptor (*Ar*) and estrogen receptor 1 and 2 (*Esr1* and *Esr2*) as well as (F) AR target gene: glutathione peroxidase 5 (*Gpx5*), lipocalin 5 (*Lcn5*), cysteine-rich secretory protein 1 (*Crisp1*) mRNA in IS and CAP of 2 month-old control and *Dicer1* conditional knock-out (cKO) mice epididymides. Expression values relative to *L19* expression. Statistical significance was calculated from the expression levels of 3 control and 4 *Dicer1^fl/fl^; Defb41^iCre/wt^* mouse samples using the unpaired t-test. Statistical significance of changes is indicated as follows: ns, not significant; *, *P≤0.05*; ***, *P≤0.001*.

## Discussion

At birth, the epididymis consists of a long convoluted duct lacking both cell type and segment specific markers. In the mouse epididymis, differentiation of the epithelial cell types takes place between day 14 and day 21 [32] after which segment-specific gene expression can be observed [14]. Nonetheless, very little is known about the regulatory pathways responsible for the epididymal segmentation and the differentiation of epithelial cell types. In this study, we demonstrate that post-transcriptional regulation via RNAi signaling is an important regulator in the development of the proximal epididymal segments.

In our *Dicer1^fl/fl^; Defb41^iCre/wt^* mouse model the ablation of *Dicer1* begins 12 days pp but, at the time of the final cell type differentiation during puberty, the epididymal epithelium of *Dicer1^fl/fl^; Defb41^iCre/wt^* mice still resembles that of control mice. Studies revealed a significant reduction in the expression of IS specific genes at 21 days pp, and about 2 weeks later, the epithelium began to regress. At 45 days of age, the *Dicer1* KO IS morphologically resembled that of an undifferentiated pre-pubertal epididymis. Despite the morphological changes, *Dicer1* ablation did not affect cell type differentiation as all major epithelial cell types were detected in the epididymides of 2 month-old *Dicer1^fl/fl^; Defb41^iCre/wt^* mice. However, the function of the principal cells was compromised as they displayed a significant reduction in segment specific gene expression.

When studying the different epithelial cell types, we detected a thicker layer of smooth muscle cells surrounding the duct of the *Dicer1^fl/fl^; Defb41^iCre/wt^* mouse epididymis. As *Dicer1* ablation is not likely to occur in the stromal tissue, the observed increase in smooth muscle cell number may indicate altered epithelial-mesenchymal signaling. It has previously been shown that cross talk between different cell types is essential for normal epididymal functions, and for example the communication between clear cells and other epithelial cell types is required to maintain luminal pH [33]. Interestingly, there are species-specific differences in thickness and distribution of the epididymal smooth muscle cell layer [34]–[36]. Further investigations regarding smooth muscle cell differentiation could thus benefit from comparative studies of different species.

Previous studies have shown that ROS1 is one of the master regulators of IS development, as the lack of both ROS1 and its negative regulator, protein tyrosine phosphatase SHP-1, causes a defect in IS differentiation [5], [37]. This affects also the regulation of sperm volume as the majority of sperm from the *Ros1* KO CAU present with angulated tails [38]. However, *Dicer1* cKO IS still has ∼70% of the *Ros1* expression of the control IS and the dedifferentiated IS does not cause a significant increase in sperm tail angulation. Furthermore, males with one intact *Ros1* allele have normal epididymal development [5]. These data indicate that the epididymal phenotype, observed in the present study, is independent of ROS1 signaling. Other proteins that have a regulative role in IS are the Fibroblast growth factors. They control gene expression by binding to FGFRs in the epithelium of IS [39], [40]. Studies in other male reproductive organs also indicate a role for FGFRs in regulating stromal-epithelial induction of cell proliferation and tissue homeostasis [41]. To assess the effect of FGF signaling on the observed epididymal phenotype, we studied the expression of *Fgfr*s in the epididymis. However, no changes were observed and it is therefore unlikely that FGF signaling contributes to the dedifferentiation of the epithelium or to the increased smooth muscle cell layer thickness of the *Dicer1^fl/fl^; Defb41^iCre/wt^* mice.

Sex steroids are important regulators of epididymal development and function. In the epididymis, epithelial expression of *Ar* starts around E14.5–15.5 [10], [42] and is required for IS development [7] and for the differentiation of principal and basal cells in the proximal epididymis [6], [10]. A further role for androgen signaling is observed after orchidectomy, where androgen depletion leads to extensive apoptosis throughout the epididymal epithelium of both prepubertal and postpubertal animals [43]. In male reproductive organs, estrogens are produced in the testis, spermatozoa and epididymis [44], [45]. *Esr1* is highly expressed throughout the efferent ducts, where it regulates fluid reabsorption of non-ciliated epithelial cells [46], [47]. Less is known about the function of ESR1 in the epididymis, although high expression of *Esr1* is detected in the narrow cells of IS and throughout the epithelium of CAP [46]. Recent studies have indicated a role for ESR1 in luminal pH maintenance [48], [49] as well as in smooth muscle contractility [50]. *Esr2* is transcribed in all cells of the epithelium, however, its role in the epididymis is still unknown since the full knock-out of *Esr2* does not display any difference in morphology or function of the epididymis compared with that of WT mice [51].

The *Dicer1^fl/fl^; Defb41^iCre/wt^* mouse model presents with a significant downregulation in *Ar*, known AR target genes and *Esr2* mRNA expression in the proximal epididymis. However, on the basis of the current study, we cannot distinguish whether the down-regulation of the target genes is due to a direct lack of AR signaling or an effect of the observed epithelial cell dedifferentiation. ESR1, on the other hand, displayed an altered expression pattern in the *Dicer1* KO IS, with expression not only in narrow cells, as in IS of control mice, but throughout the epithelium. As ESR1 is known to promote cell proliferation [52], high ESR1 levels in the *Dicer1* cKO IS could explain the observed increase in cell proliferation in that epididymal segment.

Studies on excessive estrogen signaling in males suggest that an imbalance in the testosterone-estrogen ratio could be of more importance than the actual increase in estrogen signaling [53]. Administration of a single high dose of diethylstilbestrol (DES), a potent synthetic estrogen, to neonatal rats, caused a reduction in the epithelial height of the epididymis. However, the increased estrogen levels gave rise to an additional downregulation of AR in the entire epididymis and thus an imbalance in sex steroid signaling. When the estrogen-testosterone ratio was adjusted by co-administration of testosterone, no phenotypic abnormalities were observed [54]. The reduction in *Ar* expression observed in *Dicer1^fl/fl^; Defb41^iCre/wt^* mice could therefore not only have significant consequences for epididymal development but also be directly caused by the increased ESR1 levels. However, a recent study on a proximal epididymis-specific AR cKO, also showed ESR1 expression in all cell types of the proximal epididymal epithelium [7]. This would indicate a role for AR in the repression of *Esr1* expression. AR cKO epididymides also showed similar features to those of the *Dicer1^fl/fl^; Defb41^iCre/wt^* mice with an altered epididymal stroma showing a disrupted smooth muscle cell layer or a thickened mesenchyme [7], [55]. In light of these studies, AR and ESR1 expression in the epididymis seem to be tightly linked and although our current results show an imbalance in sex steroid receptor ratio we cannot with certainty say if the imbalance was initiated by a lowered AR expression or an increase in ESR1 expression.

Adams et al. were the first to show that *Esr1* is a direct target of miR-206 [56]. Since then, around 14 evolutionarily conserved miRNAs have been found to target directly the 3′-UTR of mammalian *Esr1* [57]. There is also evidence of a miRNA-induced downregulation of *Esr1* after testosterone treatment in the female mouse liver [58]. In light of these previous studies the upregulation of ESR1 in *Dicer1* KO IS might be directly ascribable to the ablation of mature miRNAs. Interestingly, previous studies have shown induction of miRNA expression by AR *in vivo* [59]. Androgen Responsive Elements (AREs) are also found in the promoter region of several miRNAs [60], [61]. Although AR would not induce expression of all epididymal miRNAs, the observed phenotype of *Dicer1^fl/fl^; Defb41^iCre/wt^* mice could be partially AR dependent. To understand better the role of the RNAi pathway during epididymal development, miRNA expression data from different developmental time points is needed. These results could be further compared to already available array data from the human and rat epididymis [27], [28] to demonstrate evolutionarily conserved RNAi regulation.

In conclusion, the present study shows the importance of the RNAi pathway in the postnatal development of the proximal epididymis. Ablation of *Dicer1* in the epididymal epithelium causes a regression in cell differentiation. The epithelium maintains cell type identity but lacks a segment-specific gene expression pattern. Furthermore, the epididymal phenotype of *Dicer1^fl/fl^; Defb41^iCre/wt^* mice resembles that of mice with an imbalanced ratio of sex steroid receptors, AR cKO mice and mice with excess estrogen signaling. This suggests that Dicer1-dependent pathways are important regulators of balanced estrogen-androgen action in the mouse epididymis.

## Materials and Methods

### Ethics Statement

All mice were handled in accordance with the institutional animal care policies of the University of Turku (Turku, Finland), and every effort was made to minimize suffering of the animals. Mice were specific pathogen-free, fed with complete pelleted chow and tap water *ad libitum* in a room with controlled light (12 hours light, 12 hours darkness) and temperature (21±1°C). Animal experiments were approved by the Finnish Animal Ethics Committee (license number: 20072-Sipilä and 2010-05926-Poutanen), and the institutional policies on animal experimentation fully met the requirements as defined in the NIH Guide on animal experimentation.

### Experimental Animals

To inactivate *Dicer1* conditionally from the epithelial cells of the proximal epididymis, *Dicer1^fl/fl^* mice [29] were crossed with a heterozygous *Defb41^iCre^* knock-in (KI) mouse line. The KI was generated by inserting the *iCre* cDNA allelle into the translation initiation site of the *Defb41* gene. A detailed characterization of the *Defb41^iCre^* KI mouse line will be described elsewhere. *Defb41^iCre/wt^* mice were genotyped with the primers Defb41 Fw: TCCATTGCCTTTTCTTGTCC and Defb41 Re: TTGTCTTACCAGGTTTCCTCCT, Tm 56°C, for the wild-type (WT) allele and iCre Fw: TCTCCAACCTGCTGACTGTG and iCre Re: AGGGACACAGCATTGGAGTC, Tm 59°C, for the iCre allele. *Dicer^fl/fl^* mice were genotyped as previously described [29]. For detection of the floxed allele, IS together with CAP, COR together with CAU and testes from 12 day-old mice were collected. After DNA extraction, the recombined allele was detected by genomic PCR as previously described [29]. *Dicer1^fl/fl^; Defb41^iCre/wt^* mice and the controls, *Dicer1^fl/fl^* mice, were obtained from the same litters. The genetic background of these mice was mixed C57Bl/6N and SV129.

### Sperm Morphology and Fertility of *Dicer^fl/fl^; Defb41^iCre/wt^* Mice

To study male fertility, 2–3 month-old male *Dicer1^fl/fl^; Defb41^iCre/wt^* mice and control mice were mated with FVB/N female mice. The female mice were superovulated by intraperitoneal injections of 5 IU pregnant mare serum gonadotropin (PMSG, NHPP, Dr. Parlow) and 5 IU human chorionic gonadotrophin (hCG, Pregnyl, Schering-Plough) 26 and 2 hours before mating, respectively. Females were checked for copulatory plugs the following morning. The mated females were followed for 3–4 weeks to determine the number of litters and offspring produced by each male. For sperm analyses, CAU of two-month-old control and *Dicer1^fl/fl^; Defb41^iCre/wt^* mice were dissected and incubated in 300 µl of HTF medium (William A. Cook Australia Pty. Ltd, Brisbane, Australia) at 37°C, 5% CO2 for 30 min, to allow the sperm to swim out. The sperm were spread on microscope slides and stained using the Papanicolaou technique (Haematoxylin, OG-6 and EA-50). The morphology of 100 sperm/sample from 4 control and 5 *Dicer1^fl/fl^; Defb41^iCre/wt^* mice samples was analyzed.

### Histology, Immunohistochemistry and β-galactosidase Staining

Epididymides from 19 day - 6 month-old control and *Dicer1^fl/fl^; Defb41^iCre/wt^* mice were fixed overnight in 4% paraformaldehyde (PFA), embedded in paraffin and prepared for histological analyses by standard procedures. Haematoxylin and eosin (HE) staining was performed by standard procedures. Epithelial cell height was measured by using the Leica IM500 imaging software. To detect the different epithelial cell types of the epididymis, the slides were incubated with the following antibodies overnight at 4°C: rabbit monoclonal anti-Keratin 5 1:100 (RM-2106, Thermo Scientific), rabbit polyclonal anti-V-ATPase 1∶100 (a gift from S. Breton, Program in Membrane Biology, MGH Simches Research Center, Boston, MA) and mouse monoclonal anti-α-actin 1∶500 (sc-32251, Santa Cruz Biotechnology). All the primary antibodies were diluted in PBS supplemented with 1% bovine serum albumin. The antibody-antigen complexes were visualized by incubation for 30 min at room temperature with 1∶200 Alexa Fluor 488- and 594-conjugated goat anti-mouse and goat anti-rabbit antibodies (Invitrogen). For detection of F-actin, the epididymides of 2 month-old mice were fixed for 15 min in 4% paraformaldehyde and rapidly frozen. The frozen sections (8 µm) were immunostained with Phalloidin-TRITC 1∶400 (P1951, Sigma-Aldrich). All slides used for the above mentioned stainings were counterstained with 4′,6-diamino-2-phenylindole dihydrochloride (DAPI, Sigma) and mounted in Mowiol 4–88 (Sigma). For sex steroid receptor detection the following antibodies were used: rabbit polyclonal anti-AR (N-20) 1∶1000 (sc-816, Santa Cruz Biotechnology), mouse monoclonal anti-estrogen receptor α 1∶200 (M7040, Dako) and mouse monoclonal anti-estrogen receptor β1 1:100 (M7292, Dako). The antibody-antigen complexes were visualized by using anti-rabbit and anti-mouse HRP labeled polymer (EnVision, Dako) combined with DAB+ chromogen system (Dako).

For β-galactosidase staining two-month-old mice epididymides were fixed in 0.2% glutaraldehyde, 2 mM MgCl_2_, 5 mM EGTA in PBS for 30 minutes. The tissues were washed overnight and stained for 2 hours at 37°C in 2 mM MgCl_2_, 0.01% NaDeoxycholate, 0.02% Tergitol-type NP-40, 5 mM K_4_Fe(CN)_6_, 5 mM K_3_Fe(CN)_6_, 1 mg/ml X-gal in PBS.

### RNA Isolation and qRT-PCR

For analyzes of gene expression, 1 day – 42 day-old WT mice (mixed background, C57Bl/6N and SV129) and 21 day - 2 month-old control and *Dicer1^fl/fl^; Defb41^iCre/wt^* mice were used. The epididymides, efferent ducts and testes were dissected out and weighed. The epididymis of control and *Dicer1^fl/fl^; Defb41^iCre/wt^* mice was cut into three segments; IS and CAP, COR and CAU. All tissues were frozen in liquid nitrogen and stored at −80°C. Total RNA was isolated from the tissues using Tri Reagent according to the manufacturer’s instructions (Molecular Research Center, Inc.). For RT-PCR, 1 µg of total RNA was treated with deoxyribonuclease I (DNaseI, Amplification Grade, Invitrogen) and reverse-transcribed by using the DyNAmo cDNA synthesis kit (Thermo Scientific). The cDNA was diluted 1∶50–1∶100 for quantitative PCR. Quantitative PCR was performed using the DyNAmo Flash SYBR Green qPCR Kit (Thermo Scientific). All samples were run in triplicate reactions and standards in duplicate reactions. *L19* and *Ppia* were used as endogenous controls to equalize for the amounts of RNA in the epididymis and testis, respectively. Primer sequences and qRT-PCR conditions for analyzing the expression of *Dicer1*, *Ros1*, *Bmyc*, *Lcn8*, *Cst8*, *Gpr64*, *Ar*, *Esr1*, *Esr2*, *Gpx5*, *Lcn5*, *Crisp1* and *Fgfr 1–4* are described in Table S1.

### Cell Proliferation and Apoptosis

To detect cell proliferation, epididymis sections were stained with rat monoclonal anti-Ki67 1:500 (M7249, Dako). The antibody-antigen complexes were visualized by using 1∶200 rabbit polyclonal anti-rat (Dako) combined with anti-rabbit HRP-labeled polymer and DAB+ chromogen system. Terminal Uridine Deoxynucleotidyl Nick End Labeling (TUNEL) was used to detect apoptotic cells. Labeling was performed by using 0.8 U/µl TdT (Terminal transferase, recombinant, Roche) and Biotin-16-dUTP (Roche), 1 h at 37°C. The reaction was visualized with Biotin coupled with ExtrAvidine-Peroxidase, 1∶200 (Sigma-Aldrich) combined with DAB+ chromogen system. Proliferating and apoptotic cells were counted from ten tubular cross sections of 5 control and 5–8 *Dicer1^fl/fl^; Defb41^iCre/wt^* mouse IS and CAP, respectively. The number of proliferative and apoptotic cells was then divided by the circumference (mm) of the tubular cross sections.

### Statistical Analyses

For statistical analyses of organ weights, epithelial cell heights, animal fertility, sperm morphology, cell proliferation/apoptosis and qRT-PCR, the GraphPad Prism 5 software (GraphPad Software, Inc., La Jolla, CA) was used. Unpaired t-test was used to determine statistical significances, assigning *P≤0.05* as the limit of statistical significance.

## Supporting Information

Figure S1 **Changes in the epithelium of the efferent ducts.** Hematoxylin and eosin staining of efferent ducts of six-month-old (A) control and (B) *Dicer1^fl/fl^; Defb41^iCre/wt^* mice. Arrow marks neoplastic changes. Scale bar 100 µm.
(TIF)

Figure S2 **Varied phenotype of the Dicer1 cKO initial segment.** Haematoxylin and eosin staining of (A) initial segment (IS) and (D) corpus (COR) of a 2 month-old control mouse and (B, C) IS of two individual 2 month-old Dicer1fl/fl; Defb41iCre/wt mice. Scale bar 100 µm.
(TIF)

Figure S3 **Segment-specific gene expression during epididymal development.** Expression of proximal epididymis specific genes in the initial segment and caput of 21 days – two-month-old control and Dicer1 conditional knock-out (cKO) mice. Expression of (A) cystatin 8 (Cst8), (B) lipocalin 8 (Lcn8) and (C) Ros1 proto-oncogene (Ros1) relative to L19 expression. Statistical significance was calculated from the expression levels of 3 control and 3 Dicer1fl/fl;Defb41iCre/wt mouse samples from each time point, using the unpaired t-test. Statistical significance of changes is indicated as follows: *, P≤0.05; **, P≤0.01; ***, P≤0.001.
(TIF)

Table S1 (DOC)

## References

[1] Clulow J, Jones RC, Hansen LA, Man SY (1998). Fluid and electrolyte reabsorption in the ductuli efferentes testis.. J Reprod.

[2] Robaire B, Syntin P, Jervis K, Jegou B (2000). The coming of age of the epididymis.. Testis, Epididymis and Technologies in the Year 2000.

[3] Cornwall GA (2009). New insights into epididymal biology and function.. Hum Reprod Update.

[4] Sipila P, Cooper TG, Yeung CH, Mustonen M, Penttinen J (2002). Epididymal dysfunction initiated by the expression of simian virus 40 T-antigen leads to angulated sperm flagella and infertility in transgenic mice.. Mol Endocrinol.

[5] Sonnenberg-Riethmacher E, Walter B, Riethmacher D, Godecke S, Birchmeier C (1996). The c-ros tyrosine kinase receptor controls regionalization and differentiation of epithelial cells in the epididymis.. Genes Dev.

[6] Krutskikh A, De Gendt K, Sharp V, Verhoeven G, Poutanen M (2011). Targeted inactivation of the androgen receptor gene in murine proximal epididymis causes epithelial hypotrophy and obstructive azoospermia.. Endocrinology.

[7] O'Hara L, Welsh M, Saunders PT, Smith LB (2011). Androgen receptor expression in the caput epididymal epithelium is essential for development of the initial segment and epididymal spermatozoa transit.. Endocrinology.

[8] Joseph A, Yao H, Hinton BT (2009). Development and morphogenesis of the Wolffian/epididymal duct, more twists and turns.. Dev Biol.

[9] Welsh M, Saunders PT, Sharpe RM (2007). The critical time window for androgen-dependent development of the wolffian duct in the rat.. Endocrinology.

[10] Murashima A, Miyagawa S, Ogino Y, Nishida-Fukuda H, Araki K (2011). Essential roles of androgen signaling in wolffian duct stabilization and epididymal cell differentiation.. Endocrinology.

[11] Tomaszewski J, Joseph A, Archambeault D, Yao HH (2007). Essential roles of inhibin beta A in mouse epididymal coiling.. Proc Natl Acad Sci U S A.

[12] Sun EL, Flickinger CJ (1979). Development of cell types and of regional differences in the postnatal rat epididymis.. Am J Anat.

[13] Hermo L, Barin K, Robaire B (1992). Structural differentiation of the epithelial cells of the testicular excurrent duct system of rats during postnatal development.. Anat Rec.

[14] Kirchhoff C (1999). Gene expression in the epididymis.. Int Rev Cytol.

[15] Mendive F, Laurent P, Van Schoore G, Skarnes W, Pochet R (2006). Defective postnatal development of the male reproductive tract in LGR4 knockout mice.. Dev Biol.

[16] Hoshii T, Takeo T, Nakagata N, Takeya M, Araki K (2007). LGR4 regulates the postnatal development and integrity of male reproductive tracts in mice.. Biol Reprod.

[17] McManus MT, Sharp PA (2002). Gene silencing in mammals by small interfering RNAs.. Nat Rev Genet.

[18] Bernstein E, Caudy AA, Hammond SM, Hannon GJ (2001). Role for a bidentate ribonuclease in the initiation step of RNA interference.. Nature.

[19] Carthew RW (2006). Gene regulation by microRNAs.. Curr Opin Genet Dev.

[20] Huang Y, Shen XJ, Zou Q, Wang SP, Tang SM (2011). Biological functions of microRNAs: A review.. J Physiol Biochem.

[21] Bernstein E, Kim SY, Carmell MA, Murchison EP, Alcorn H (2003). Dicer is essential for mouse development.. Nat Genet.

[22] Soukup GA, Fritzsch B, Pierce ML, Weston MD, Jahan I (2009). Residual microRNA expression dictates the extent of inner ear development in conditional dicer knockout mice.. Dev Biol.

[23] Muljo SA, Ansel KM, Kanellopoulou C, Livingston DM, Rao A (2005). Aberrant T cell differentiation in the absence of dicer.. J Exp Med.

[24] Lynn FC, Skewes-Cox P, Kosaka Y, McManus MT, Harfe BD (2007). MicroRNA expression is required for pancreatic islet cell genesis in the mouse.. Diabetes.

[25] Sekine S, Ogawa R, Mcmanus MT, Kanai Y, Hebrok M (2009). Dicer is required for proper liver zonation.. J Pathol.

[26] McKenna LB, Schug J, Vourekas A, McKenna JB, Bramswig NC (2010). MicroRNAs control intestinal epithelial differentiation, architecture, and barrier function.. Gastroenterology 139(5): 1654–64,.

[27] Zhang J, Liu Q, Zhang W, Li J, Li Z (2010). Comparative profiling of genes and miRNAs expressed in the newborn, young adult, and aged human epididymides.. Acta Biochim Biophys Sin (Shanghai).

[28] Wang J, Ruan K (2010). miR-335 is involved in the rat epididymal development by targeting the mRNA of RASA1.. Biochem Biophys Res Commun.

[29] Harfe BD, McManus MT, Mansfield JH, Hornstein E, Tabin CJ (2005). The RNaseIII enzyme dicer is required for morphogenesis but not patterning of the vertebrate limb.. Proc Natl Acad Sci U S A.

[30] Yuan Q, Guo QS, Cornwall GA, Xu C, Wang YF (2007). Age-dependent expression of the cystatin-related epididymal spermatogenic (cres) gene in mouse testis and epididymis.. Asian J Androl.

[31] Lareyre JJ, Winfrey VP, Kasper S, Ong DE, Matusik RJ (2001). Gene duplication gives rise to a new 17-kilodalton lipocalin that shows epididymal region-specific expression and testicular factor(s) regulation.. Endocrinology.

[32] Avram CE, Cooper TG (2004). Development of the caput epididymidis studied by expressed proteins (a glutamate transporter, a lipocalin and beta-galactosidase) in the c-ros knockout and wild-type mice with prepubertally ligated efferent ducts.. Cell Tissue Res.

[33] Shum WW, Ruan YC, Da Silva N, Breton S (2011). Establishment of cell-cell crosstalk in the epididymis: Control of luminal acidification..

[34] Abd-Elmaksoud A (2009). Comparative expression of laminin and smooth muscle actin in the testis and epididymis of poultry and rabbit.. J Mol Histol.

[35] Alkafafy M, Elnasharty M, Sayed-Ahmed A, Abdrabou M (2011). Immunohistochemical studies of the epididymal duct in egyptian water buffalo (bubalus bubalis).. Acta Histochem.

[36] Egger G, Witter K (2009). Peritubular contractile cells in testis and epididymis of the dog, canis lupus familiaris..

[37] Keilhack H, Muller M, Bohmer SA, Frank C, Weidner KM (2001). Negative regulation of ros receptor tyrosine kinase signaling. an epithelial function of the SH2 domain protein tyrosine phosphatase SHP-1.. J Cell Biol.

[38] Yeung CH, Sonnenberg-Riethmacher E, Cooper TG (1999). Infertile spermatozoa of c-ros tyrosine kinase receptor knockout mice show flagellar angulation and maturational defects in cell volume regulatory mechanisms.. Biol Reprod.

[39] Lan ZJ, Labus JC, Hinton BT (1998). Regulation of gamma-glutamyl transpeptidase catalytic activity and protein level in the initial segment of the rat epididymis by testicular factors: Role of basic fibroblast growth factor.. Biol Reprod.

[40] Kirby JL, Yang L, Labus JC, Hinton BT (2003). Characterization of fibroblast growth factor receptors expressed in principal cells in the initial segment of the rat epididymis.. Biol Reprod.

[41] Cotton LM, O'Bryan MK, Hinton BT (2008). Cellular signaling by fibroblast growth factors (FGFs) and their receptors (FGFRs) in male reproduction.. Endocr Rev.

[42] Crocoll A, Zhu CC, Cato AC, Blum M (1998). Expression of androgen receptor mRNA during mouse embryogenesis.. Mech Dev.

[43] Takagi-Morishita Y, Kuhara A, Sugihara A, Yamada N, Yamamoto R (2002). Castration induces apoptosis in the mouse epididymis during postnatal development.. Endocr J.

[44] Carreau S, Genissel C, Bilinska B, Levallet J (1999). Sources of oestrogen in the testis and reproductive tract of the male.. Int J Androl.

[45] Shayu D, Rao AJ (2006). Expression of functional aromatase in the epididymis: Role of androgens and LH in modulation of expression and activity.. Mol Cell Endocrinol.

[46] Zhou Q, Nie R, Prins GS, Saunders PT, Katzenellenbogen BS (2002). Localization of androgen and estrogen receptors in adult male mouse reproductive tract.. J Androl.

[47] Hess RA, Bunick D, Lubahn DB, Zhou Q, Bouma J (2000). Morphologic changes in efferent ductules and epididymis in estrogen receptor-alpha knockout mice.. J Androl.

[48] Joseph A, Hess RA, Schaeffer DJ, Ko C, Hudgin-Spivey S (2010). Absence of estrogen receptor alpha leads to physiological alterations in the mouse epididymis and consequent defects in sperm function.. Biol Reprod.

[49] Joseph A, Shur BD, Ko C, Chambon P, Hess RA (2010). Epididymal hypo-osmolality induces abnormal sperm morphology and function in the estrogen receptor alpha knockout mouse.. Biol Reprod.

[50] Fibbi B, Filippi S, Morelli A, Vignozzi L, Silvestrini E (2009). Estrogens regulate humans and rabbit epididymal contractility through the RhoA/Rho-kinase pathway.. J Sex Med.

[51] Rosenfeld CS, Ganjam VK, Taylor JA, Yuan X, Stiehr JR (1998). Transcription and translation of estrogen receptor-beta in the male reproductive tract of estrogen receptor-alpha knock-out and wild-type mice.. Endocrinology.

[52] Morani A, Warner M, Gustafsson JA (2008). Biological functions and clinical implications of oestrogen receptors alfa and beta in epithelial tissues.. J Intern Med.

[53] Williams K, McKinnell C, Saunders PT, Walker M, Fisher JS (2001). Neonatal exposure to potent and environmental oestrogens and abnormalities of the male reproductive system in the rat: Evidence for importance of the androgen-oestrogen balance and assessment of the relevance to man.. Hum Reprod Update.

[54] McKinnell C, Atanassova N, Williams K, Fisher JS, Walker M (2001). Suppression of androgen action and the induction of gross abnormalities of the reproductive tract in male rats treated neonatally with diethylstilbestrol.. J Androl.

[55] Simanainen U, McNamara K, Davey RA, Zajac JD, Handelsman DJ (2008). Severe subfertility in mice with androgen receptor inactivation in sex accessory organs but not in testis.. Endocrinology.

[56] Adams BD, Furneaux H, White BA (2007). The micro-ribonucleic acid (miRNA) miR-206 targets the human estrogen receptor-alpha (ERalpha) and represses ERalpha messenger RNA and protein expression in breast cancer cell lines.. Mol Endocrinol.

[57] Pandey DP, Picard D (2010). Multidirectional interplay between nuclear receptors and microRNAs.. Curr Opin Pharmacol.

[58] Delic D, Grosser C, Dkhil M, Al-Quraishy S, Wunderlich F (2010). Testosterone-induced upregulation of miRNAs in the female mouse liver.. Steroids.

[59] Narayanan R, Jiang J, Gusev Y, Jones A, Kearbey JD (2010). MicroRNAs are mediators of androgen action in prostate and muscle.. PLoS One.

[60] Ribas J, Ni X, Haffner M, Wentzel EA, Salmasi AH (2009). miR-21: An androgen receptor-regulated microRNA that promotes hormone-dependent and hormone-independent prostate cancer growth.. Cancer Res.

[61] Shi XB, Xue L, Yang J, Ma AH, Zhao J (2007). An androgen-regulated miRNA suppresses Bak1 expression and induces androgen-independent growth of prostate cancer cells.. Proc Natl Acad Sci U S A.

