# Supplementary figures and images for: Dicer1 Ablation in the Mouse Epididymis Causes Dedifferentiation of the Epithelium and Imbalance in Sex Steroid Signaling
Source: PLoS One. 2012 Jun 6;7(6):e38457. doi: 10.1371/journal.pone.0038457 (PMC3368854; doi:10.1371/journal.pone.0038457)

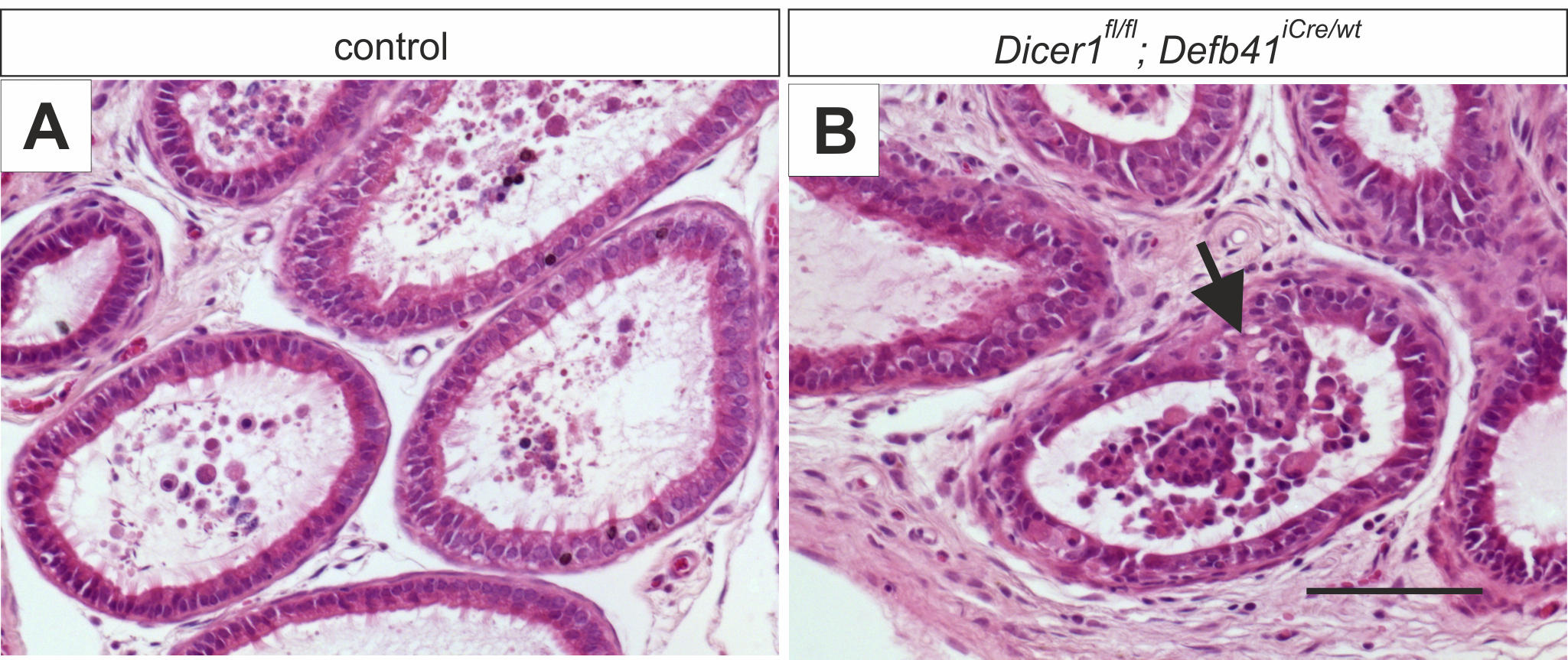

Supplement: Figure S1 — Changes in the epithelium of the efferent ducts. Hematoxylin and eosin staining of efferent ducts of six-month-old (A) control and (B) Dicer1fl/fl; Defb41iCre/wt mice. Arrow marks neoplastic changes. Scale bar 100 µm. (TIF) [file pone.0038457.s001.tif]

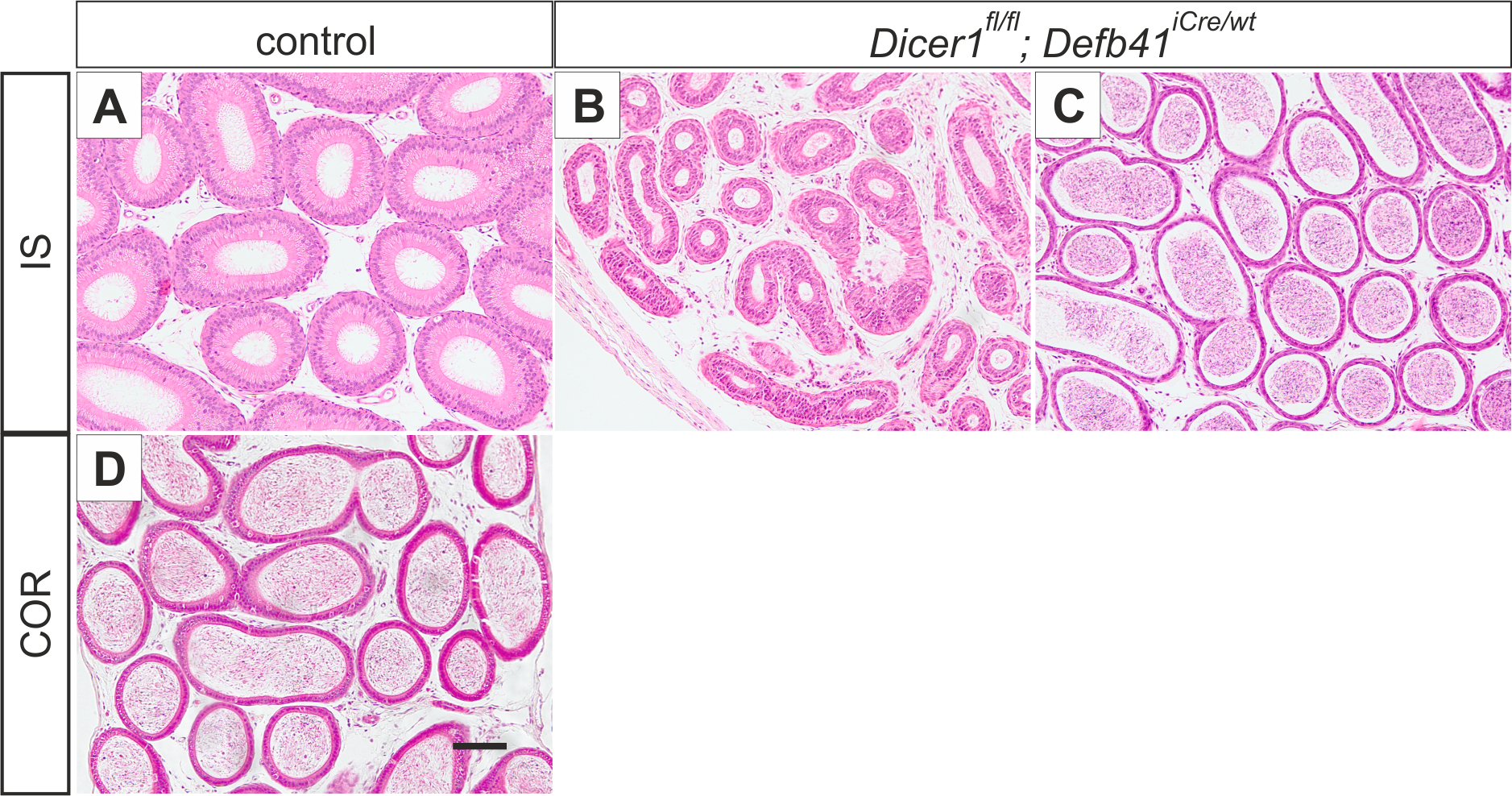

Supplement: Figure S2 — Varied phenotype of the Dicer1 cKO initial segment. Haematoxylin and eosin staining of (A) initial segment (IS) and (D) corpus (COR) of a 2 month-old control mouse and (B, C) IS of two individual 2 month-old Dicer1fl/fl; Defb41iCre/wt mice. Scale bar 100 µm. (TIF) [file pone.0038457.s002.tif]

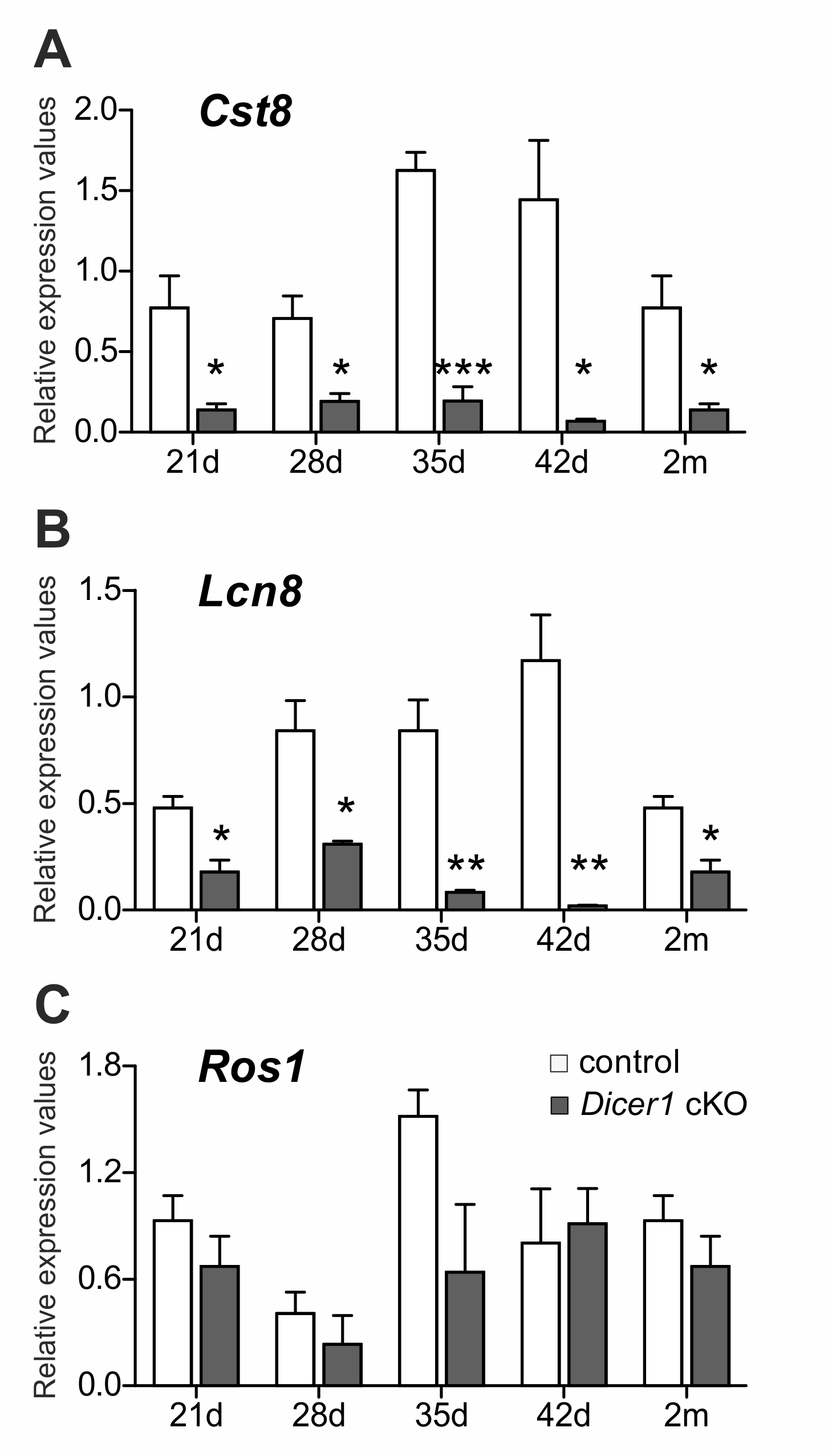

Supplement: Figure S3 — Segment-specific gene expression during epididymal development. Expression of proximal epididymis specific genes in the initial segment and caput of 21 days – two-month-old control and Dicer1 conditional knock-out (cKO) mice. Expression of (A) cystatin 8 (Cst8), (B) lipocalin 8 (Lcn8) and (C) Ros1 proto-oncogene (Ros1) relative to L19 expression. Statistical significance was calculated from the expression levels of 3 control and 3 Dicer1fl/fl;Defb41iCre/wt mouse samples from each time point, using the unpaired t-test. Statistical significance of changes is indicated as follows: *, P≤0.05; **, P≤0.01; ***, P≤0.001. (TIF) [file pone.0038457.s003.tif]
