# Supplementary material for: Dicer1 Ablation in the Mouse Epididymis Causes Dedifferentiation of the Epithelium and Imbalance in Sex Steroid Signaling
Source: PLoS One. 2012 Jun 6;7(6):e38457. doi: 10.1371/journal.pone.0038457 (PMC3368854; doi:10.1371/journal.pone.0038457)
Supplement: Table S1 — (DOC) [file pone.0038457.s004.doc]

**Table S1. Primers for qPCR**

| Genes | Primer sequences 5’>3’ | | Tm ⁰C |
| --- | --- | --- | --- |
|  | Forward | Reverse |  |
| *L19* | CTGAAGGTCAAAGGGAATGTG | GGACAGAGTCTTGATGATCTC | 60 ⁰C |
| *Ppia* | TCCATGGCTTCCACAATGTT | CATCCTAAAGCATACAGGTCCTG | 63 ⁰C |
| *Dicer1* | CTTGACTGACTTGCGCTCTG | AATGGCACCAGCAAGAGACT | 60 ⁰C |
| *Ros1* | TCTCGAGAGGCCCTTATTCA | TCACAGCCATGATGAATGGT | 59 ⁰C |
| *Bmyc* | TGGACTATGACTCGGTGCAC | CAGGTCTGCGATGGAGAACT | 56 ⁰C |
| *Ar* | GTCTCCGGAAATGTTATGAA | AAGCTGCCTCTCTCCAAG | 60 ⁰C |
| *Esr1* | TTGACAAGAACCGGAGGAAG | ATAGATCATGGGCGGTTCAG | 60 ⁰C |
| *Esr2* | GACAAGAACCGGCGTAAAAG | GGACGGCTCACTAGCACATT | 60 ⁰C |
| *Lcn8,Cst8, Gpr64* | Sipilä, P. 2002 | |  |
| *Gpx5,Lcn5,Crisp1* | Sipilä, P. 2002 | |  |
| *Fgfr 1-4* | Kurosu, H. 2007 | |  |

*Ros1*, ros1 proto-oncogene; *Bmyc*, brain expressed myelocytomatosis oncogene; Ar, androgen receptor; *Esr1*, estrogen receptor 1; *Esr2*, estrogen receptor 2; *Lcn8*, lipocalin 8; *Cst8*, cystatin 8; *Gpr64*, G protein-coupled receptor 64; *Gpx5*, glutathione peroxidase 5; *Lcn5*, lipocalin 5; *Crisp1*, cysteine-rich secretory protein 1 and *Fgfr 1-4*, fibroblast growth factor receptors 1-4.
